# Uncoupling of *in-vitro* identity of embryonic limb derived skeletal progenitors and their *in-vivo* bone forming potential

**DOI:** 10.1038/s41598-019-42259-x

**Published:** 2019-04-08

**Authors:** Louca Verbeeck, Liesbet Geris, Przemko Tylzanowski, Frank P. Luyten

**Affiliations:** 1 Prometheus, Div of Skeletal Tissue Engineering, KU Leuven, Leuven, Belgium; 2 Tissue Engineering laboratory, SBERC, KU Leuven, Leuven, Belgium; 3 Development & Stem Cell Biology laboratory, SBERC, KU Leuven, Leuven, Belgium; 4 Biomechanics Research Unit, University of Liege, Liege, Belgium; 5 Biomechanics Section, KU Leuven, Leuven, Belgium; 6 Dept of Bioch. & Mol Biol., Medical University Lublin, Lublin, Poland

## Abstract

The healing of large bone defects remains a major unmet medical need. Our developmental engineering approach consists of the *in vitro* manufacturing of a living cartilage tissue construct that upon implantation forms bone by recapitulating an endochondral ossification process. Key to this strategy is the identification of the cells to produce such cartilage intermediates efficiently. We applied a cell selection strategy based on published skeletal stem cell markers using mouse embryonic limb cartilage as cell source and analysed their potential to form bone in an *in vivo* ectopic assay. FGF2 supplementation to the culture media for expansion blocked dedifferentiation of the embryonic cartilage cells in culture and enriched for stem cells and progenitors as quantified using the recently published CD marker set. However, when the stem cells and progenitors were fractionated from expanded embryonic cartilage cells and assessed in the ectopic assay, a major loss of bone forming potential was observed. We conclude that cell expansion appears to affect the association between cell identity based on CD markers and *in vivo* bone forming capacity.

## Introduction

Large bone defects can be caused by major trauma, infection, prosthetic revision, bone tumour resection or non-healing fractures and in clinical practice, their healing remains a therapeutic challenge. Current treatments such as iliac crest autografts or cadaver allografts require multiple and repetitive interventions and are associated with various risks resulting in a high socio-economic burden^1–3^. Several tissue engineering strategies have been developed to overcome these challenges and one of them is based on bone developmental engineering. This approach involves the *in vitro* manufacturing of a living cartilage tissue construct that upon implantation forms bone *in vivo* by recapitulating endochondral ossification taking place during embryonic development.

Briefly, during that process, Prrx1 expressing limb mesenchymal cells condense and differentiate into Sox9^+^ chondrocytes. These chondrocytes proliferate, organize in columns and enter hypertrophy under the control of an Ihh/PTHrP loop. After cell maturation into Runx2^+^ hypertrophic chondrocytes, a shift in matrix synthesis occurs from collagen type II to type X. This matrix calcifies and is replaced by bone by invading osteoblasts and transdifferentiating non-apoptotic hypertrophic chondrocytes, both characterized by Osterix expression and secretion of osteoid matrix^4^.

The cell sources to engineer cartilage intermediates can be diverse with the periosteum currently considered an excellent cell source^5^. Lineage tracing experiments in mice have shown that during bone repair, osteoblasts and osteoclasts originated from the bone marrow, endosteum and periosteum, but that callus chondrocytes were primarily derived from the periosteum^6^. More recently, it has been shown that human periosteal cells can be primed *in vitro*, by using conditioned medium and cell aggregation, to a cartilaginous intermediate tissue able to develop into bone ectopically and facilitate healing in an orthotopic long bone defect^7^. However, these cells still generate excessive fibrous tissue. Enrichment for the osteochondrogenic precursors is expected to result in an enhanced bone forming potential and improved purity of cell based treatments.

Several studies in mice focused on the identification and contribution of skeletal stem cells and osteochondroprogenitors in bone development, homeostasis and fracture healing. These cells are found either in the zone underneath the growth plate, blood vessel niches or the periosteum. Several molecular markers have been associated with these cells such as Nestin, Gremlin1, Leptin Receptor, Gli1 and Periostin^8–12^. In another study, “rainbow” mice displayed a high frequency of clonal regions in the growth plate of adult mice, characterized by Alpha V Integrin (CD51) expression but negative for CD45 and TER119^13^. This population was subsequently divided into eight subpopulations based on differential expression of CD105, CD90.2, CD200 and 6C3 cell surface markers. By combining this strategy with *in vivo* and *in vitro* approaches, they mapped bone, cartilage and stromal development from a postnatal mouse skeletal stem cell to its downstream progenitors in a hierarchical program similar to hematopoiesis^13^.

In the current study, we have optimized the prospective isolation of stem and progenitor cell populations from the mouse embryonic hind limb cartilage 14.5 dpc and studied their potential for cartilage and bone formation *in vivo*. To select the desired cell subsets, we used previously described cell surface markers^13^ and studied them separately in an established *in vivo* ectopic bone formation assay in nude mice.

We show that primary mouse embryonic cartilage cells (ECC) continue their developmental program and form a bone organoid in an *in vivo* ectopic bone forming assay. Cell tracking experiments revealed the contribution of donor cells to the osseous tissue. We then purified from the embryonic cartilage cells two cell populations, namely the mouse skeletal stem cell (mSSC) and a Pre-progenitor (PreP), a direct descendent of the mSSC, and demonstrated their bone forming potential in the ectopic assay. We showed however that their potential is heavily influenced by the hydrogel encapsulating the cells. Next, when expanding the embryonic cartilage cells *in vitro* in the presence of FGF2, a standard ligand used in stem cell expansion protocols, an enrichment for stem cells and progenitors as quantified using the CD marker set was observed. However, a major loss of *in vivo* bone formation was observed, suggesting the lack of predictive value of the markers for *in vivo* bone forming potential, when *in vitro* expansion is performed.

## Results

### Isolated embryonic cartilage cells continue their developmental program and form endochondral bone *in vivo*

**Figure 1:**
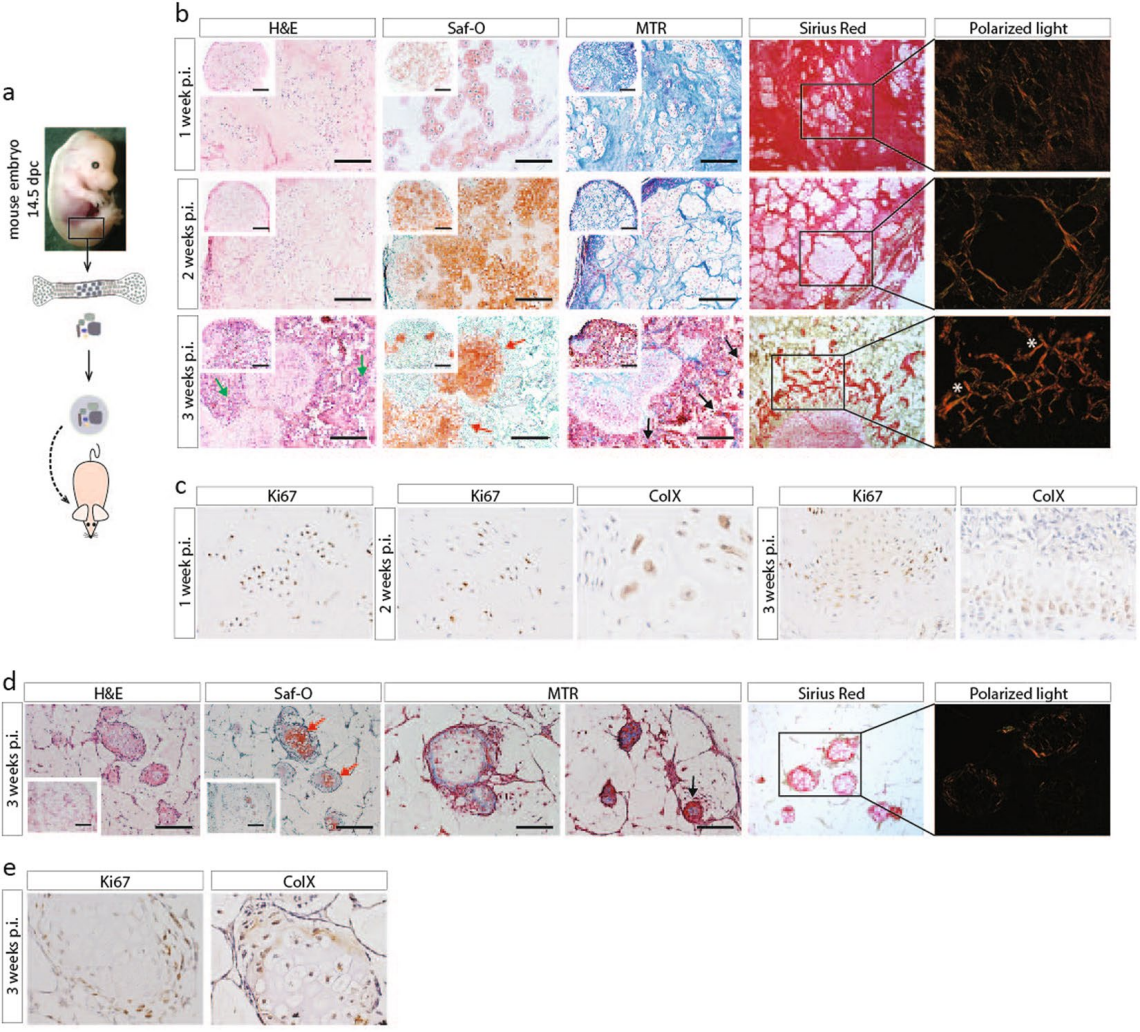
Embryonic cartilage cells are able to from bone in an adult ectopic environment through an endochondral differentiation program. (**a**) Schematic overview of experiments. ECC from 14.5dpc embryos were released by enzymatic digest and encapsulated in either collagen gel (**b,c**) or alginate (**d,e**). Gels were implanted behind the shoulders in NMRI nu/nu mice. (**b**) Histochemical analysis of explants in collagen gel one week (upper panel), two weeks (middle panel) and three weeks (lower panel) post implantation (p.i.). After three weeks *in vivo*, ossicles were retrieved. They contained sites of bone marrow formation (green arrows), growth plate-like cartilage structures (red arrows) and bone tissue comprised of osteoid matrix (black arrows) and highly organized collagen matrix (white asterix). (**c**) Immunohistochemistry for Ki67 and ColX. Positive nuclei (brown stain) were seen in one, two and three week old explants. (**d**) Histochemical analysis of explants in alginate, retrieved after three weeks. Explants contained small islets of tissue, made up of cartilage tissue (red arrows), however some osteoid secreting cells could be detected by positive red Mason’s Trichrome staining (black arrow). (**e**) Immunohistochemistry for Ki67 and ColX for alginate explants. Scale bars = 200 µm and 500 µm in the inset in (**b,d**). n = 6 for each time point.

As the first step in identifying the cell subpopulation most efficiently inducing osteochondrogenic differentiation *in vivo*, we focused on the total cell preparations from femur anlagen of 14.5 dpc mouse embryos. They were dissected and the embryonic cartilage cells were released using collagenase. In the *in vivo* bone formation assay, we used two different hydrogel encapsulation protocols, collagen type I and alginate. The latter allows for the ECC to form bone in an attachment-free environment. The cells were encapsulated in respective gels and implanted subcutaneously behind the shoulders of nude mice (Fig. 1a).

Figure 1b shows the histological analysis of collagen hydrogel based samples retrieved after one, two or three weeks. After one week, (Fig. 1b, upper panel), H&E staining showed encapsulated cells in the collagen gel, with a minimal amount of matrix synthesised as shown by the faint Safranin-O staining. Cells were proliferating as shown by positive Ki67 staining (Fig. 1c, left panel). At two weeks (Fig. 1b, middle panel), cells of various sizes were detected, ranging from small columnar and proliferative towards large, round and apoptotic, indicating maturation of cells into hypertrophy, as shown by positive ColX staining (Fig. 1c, middle panel). After three weeks *in vivo* (Fig. 1b, lower panel), the samples developed into a bone ossicle, containing trabecular bone, comprising of osteoid matrix, as shown by red Mason’s Trichrome staining. This bone tissue was associated with bone marrow formation, and islands of Safranin-O positive cartilage could still be detected in the explants. This cartilage tissue displayed growth plate zonation, with proliferative and hypertrophic chondrocytes as shown by positive staining for Ki67 and ColX (Fig. 1c, right panel). Polarized light microscopy of Sirius Red stained sections showed the presence of highly organized thick collagen fibres in the newly formed bone matrix.

Alginate encapsulated samples retrieved after three weeks revealed small islands of cartilage tissue, indicated by Safranin-O staining (Fig. 1d). Some cells in the islands were hypertrophic, as shown by positive ColX staining (Fig. 1e). As no blood vessels were observed throughout the alginate gel, the presence of invading osteoprogenitors was limited, but surprisingly, Mason’s Trichrome staining showed the presence of osteoid matrix (Fig. 1d). This led to the hypothesis that some of the cells had differentiated to osteoid producing osteoblasts. Collagen or alginate gels without cells served as negative control and no cartilage or bone formation was detected in them (see Supplementary Fig. S1).

Overall, we can conclude that embryonic cartilage cells can form bone along the endochondral ossification pathway, when encapsulated in a collagen I hydrogel. Bone formation of the cells in alginate was however limited to non-detectable.

### Donor cells differentiate into osteoblasts and contribute to bone ossicles

One of the recurring issues when investigating newly formed tissues in explants is their host or donor origin. To address that, we used ECC derived from ACTb-eGFP (ubiquitous eGFP expression) which were encapsulated in collagen hydrogel and implanted subcutaneously (see Supplementary Fig. S2). EGFP^+^ cells were tracked after one, two or three weeks by performing an immunofluorescent staining. Additional immunofluorescent staining for Sox9, Runx2 and Osterix was used to determine their differentiation state in an endochondral ossification program.

In explants retrieved after one week, eGFP^+^ cells expressing Sox9 and Runx2 were found throughout the explant, indicating cell specification of the implanted cells in the chondrogenic lineage. Similar results were observed in explants retrieved after two weeks. After three weeks, the expression of both transcription factors was confined to the cartilaginous tissues embedded in the bone organoid. Osterix, a pre-osteoblastic marker, was expressed in hypertrophic chondrocytes, similarly to Runx2, and on the cartilage-bone boundary. Past this perimeter, into the osseous tissue, we could observe eGFP^+^ donor cells expressing Osterix.

In summary, the donor-derived cells are capable of chondrogenic maturation. Furthermore, some donor cells did differentiate into osteoblasts as shown by the presence of positive eGFP Osterix expressing cells in the osseous tissue of the explants, thereby confirming donor cells contributing to the observed bone.

### Identification of mouse Skeletal Stem Cells and Pre-progenitors in embryonic limb cartilage

**Figure 2:**
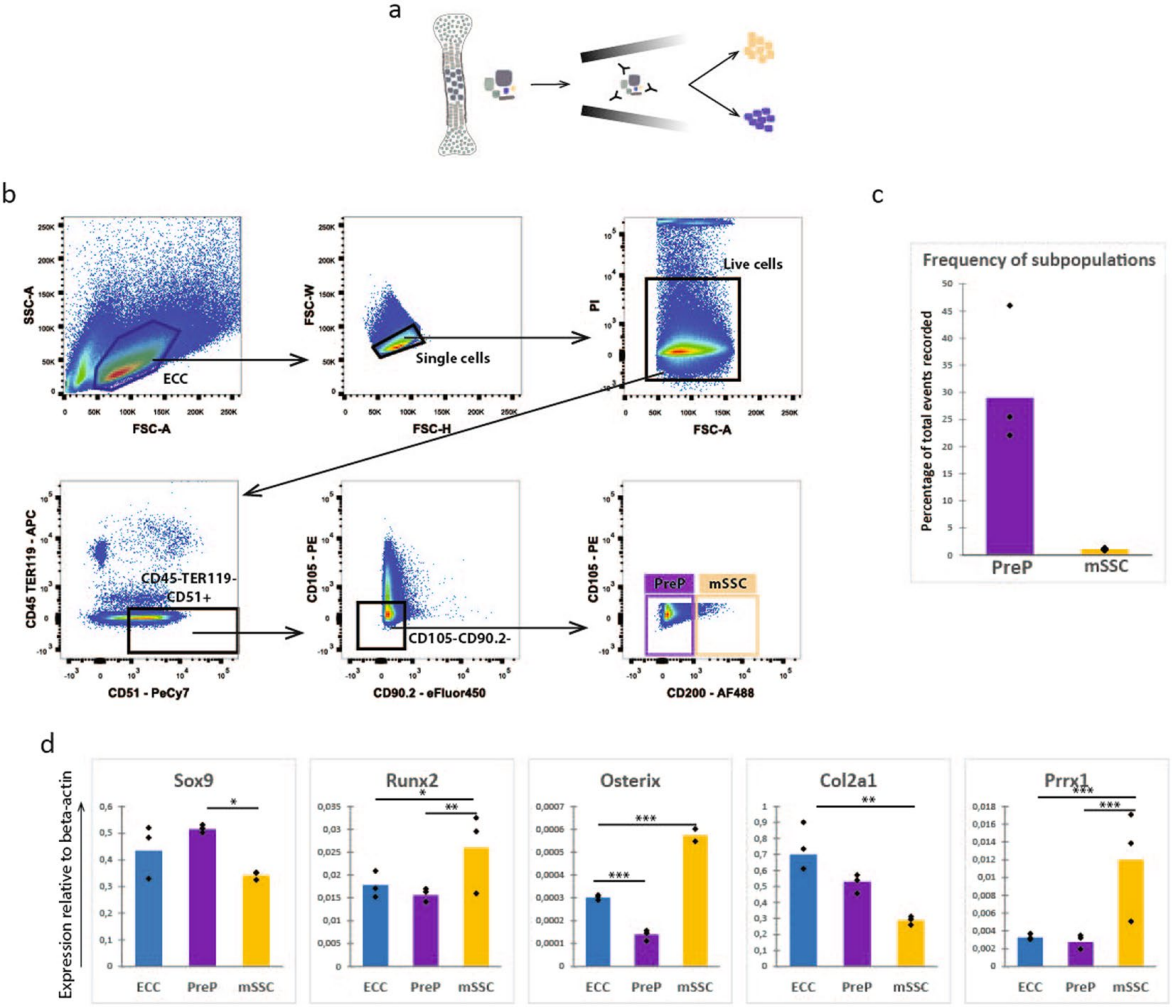
Progenitor populations can be found in the embryonic cartilage cells. (**a**) Schematic overview of experiment. ECC were stained with fluorochrome-conjungated antibodies and subjected to FACS sorting. Both populations were submitted to gene expression analysis. (**b**) Flow cytometry pseudocolour plots showing the gating strategy. Gating occurred as followed: (1) ECC in FSC-A vs SSC-A, (2) singlets in FSC-W vs FSC-H, (3) live cell selection in PI vs FSC-A, (4) CD51^+^and CD45^−^TER119^−^, (5) CD105^−^ and CD90.2^−^, (6) mSSC: CD200^+^ and PreP CD200^−^. (**c**) Graphical representation of the percentages of CD45^−^TER119^−^CD51^+^CD105^−^CD90.2^−^CD200^+^ mSSC and CD45^−^TER119^−^CD51^+^CD105^−^CD90.2^−^CD200^−^ PreP at passage zero, relative to total events recorded. (**d**) Q-PCR analysis of mRNA transcript levels for endochondral markers. Statistical analysis for significance was performed with 1 way ANOVA with Bonferroni post-hoc correction. n = 3, *p < 0.05, **p < 0.01, ***p < 0.001.

Next, we sought to investigate the presence of subsets of cells within the embryonic cartilage. The ECC were released from the limb cartilage by enzymatic digestion and isolated by fluorescent activated cell sorting (Fig. 2a). Specifically, we purified two cell populations: CD51^+^CD45^−^TER119^−^CD105^−^CD90.2^−^CD200^−^ pre–progenitors (PreP) and CD51^+^CD45^−^TER119^−^CD105^−^CD90.2^−^CD200^+^ skeletal stem cells (mSSC) (Fig. 2b). The mSSC accounted for 1,06% and the PreP for 29,89% of the ECC population (Fig. 2c). Following the isolation of both cell populations, their molecular signature was investigated further.

Two sets of genes were analysed: those relevant for endochondral ossification (Fig. 2d) and to positional information along the proximal distal axis in developing limbs (see Supplementary Fig. S3). In the former case, we detected a decreased expression of Sox9 and its downstream target Col2a1, as well as an increase in Runx2 in the mSSC. Strikingly, an increased expression of Prrx1 and Osterix was observed in the mSSC compared to unsorted primary ECC and PreP. For the latter case, we detected a small increase in Hoxa10 expression in PreP and mSSC relative to unsorted ECC.

In conclusion, and as anticipated, the expression of early chondrogenic markers (Sox9, Col2a1) in mSSC was reduced, whereas the expression of early mesodermal markers (Runx2, Prrx1) was increased. We could see a significant differential expression of Hoxa10 in PreP and mSSC, one of the genes associated with limb positional information and proximal distal outgrowth and with activation of the pre-osteoblast marker Runx2.

### Sorted mSSC and PreP from embryonic cartilage are able to from bone in the adult environment

**Figure 3:**
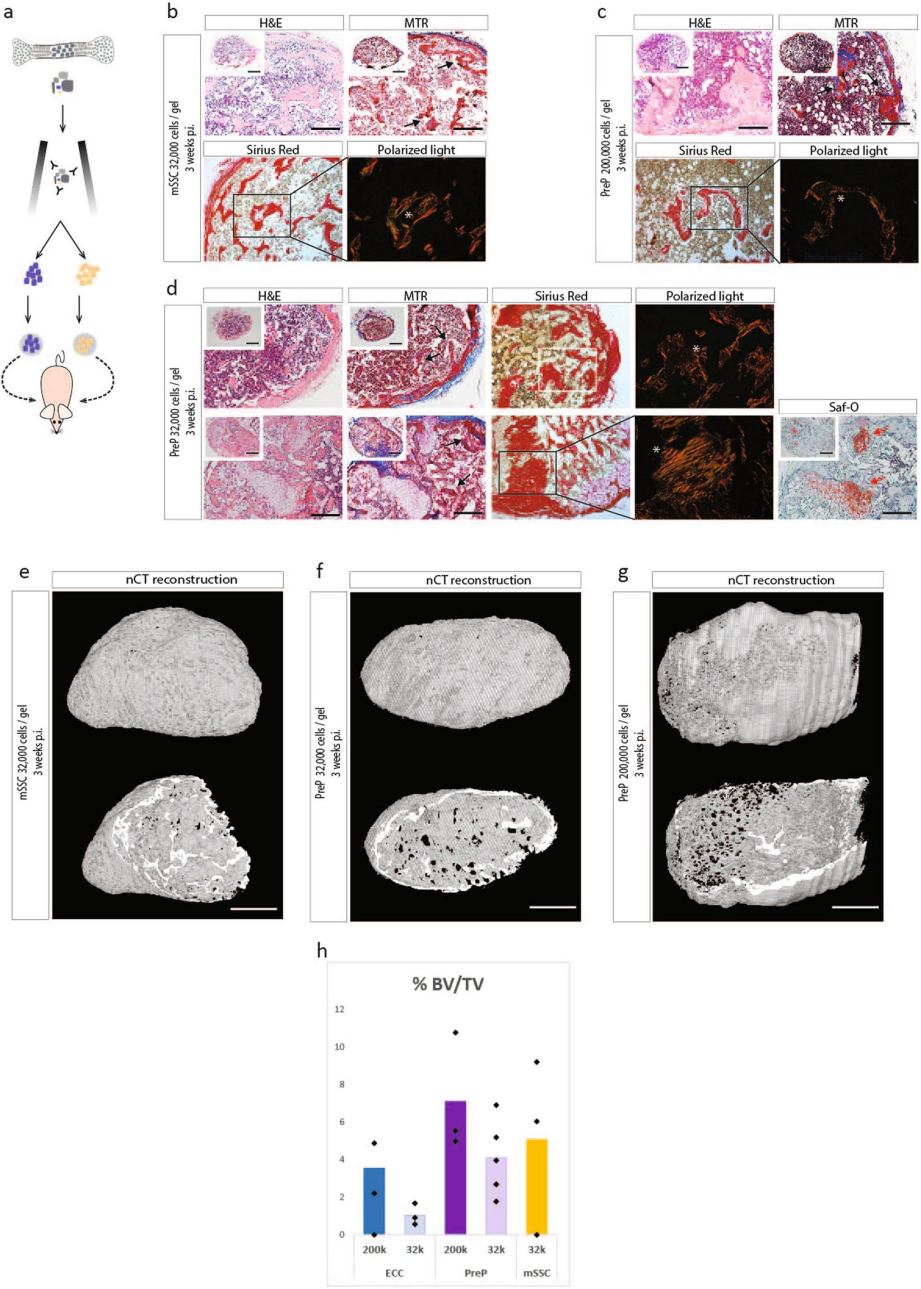
CD45^−^TER119^−^CD51^+^CD105^−^CD90.2^−^CD200^−^ PreP and CD45^−^TER119^−^CD51^+^CD105^−^CD90.2^−^CD200^+^ mSSC from embryonic cartilage are able to form bone in an ectopic *in vivo* bone formation assay. (**a**) Schematic overview of experiment. ECC were released and stained with fluorochrome-conjungated antibodies. The CD45^−^TER119^−^CD51^+^CD105^−^CD90.2^−^CD200^+^ mSSC and CD45^−^TER119^−^CD51^+^CD105^−^CD90.2^−^CD200^−^ PreP were fractioned by FACS, encapsulated in collagen I hydrogel and implanted ectopically in nude mice. (**b**–**d**) mSSC at 32,000 cells per gel (**b**) and PreP at 200,000 cells per gel (**c**) in collagen develop after three weeks in a mature ossicle with osteoid matrix (black arrows) and a highly organized collagen network (white asterix). PreP at 32,000 cells per gel also develop into bone, however in some replicates remnant cartilage tissue can be found (red arrows) (**d**). (**e**–**g**) Reconstructed images from nCT scanned explants of mSSC (**e**), PreP at 32,000 cells per gel (**f**) and PreP at 200,000 cells per gel (**g**). (**h**) Quantification of bone volume over tissue volume (% BV/TV), comparing ECC to PreP and mSSC in collagen. Scale bars = 200 µm and 500 µm in the inset in (**b,c**) 100 µm in (**e**–**g**). n = 3 for mSSC implants, n = 5 for PreP implants for *in vivo* implantation, n = 3 for bone tissue quantification.

Next, we investigated the bone forming potential of the mSSC and PreP *in vivo*, in collagen I or alginate hydrogel. The mSSC were encapsulated at a concentration of 32,000 cells and the PreP at 200,000 or 32,000 cells per implant. The hydrogels were implanted into nude mice and retrieved after three weeks *in vivo* (Fig. 3a). Nanofocus-computed tomography (nCT) showed completely mineralized bone ossicles for the collagen type I embedded explants (Fig. 3e–g). In the mSSC explants in collagen gel, histological analysis showed a bone organoid. These explants contained cortical and trabecular bone with osteoid matrix as shown by red Mason’s Trichrome staining. Collagen fibers were highly organized as shown by Picrosirius red staining and polarized light microscopy. Bone marrow was detected throughout the explant as well and cartilage was absent (Fig. 3b).

Similar bone organoids were observed when PreP were implanted at the concentration of 200,000 cells per gel (Fig. 3c). To compare better the potential of the two populations, PreP were also implanted at the same cell density as the mSSC (Fig. 3d). After three weeks *in vivo*, bone organoids could be retrieved, but some of these ossicles still had cartilage present, shown by positive Safranin-O staining (Fig. 3d, lower panel). Quantification of bone volume over tissue volume revealed a trend towards more bone formed by purified PreP and mSSC in comparison with ECC, and this for both cell densities tested (Fig. 3h). To determine whether the mSSC and PreP formed bone by the endochondral pathway, explants were retrieved after one week. Safranin-O staining indicated cartilage and immunohistochemistry staining identified ColX^+^ cells, indicating presence of matured cartilage (see Supplementary Fig. S4). This strongly suggests that the detected ossicles implanted for three weeks formed predominantly by the endochondral pathway.

**Figure 4:**
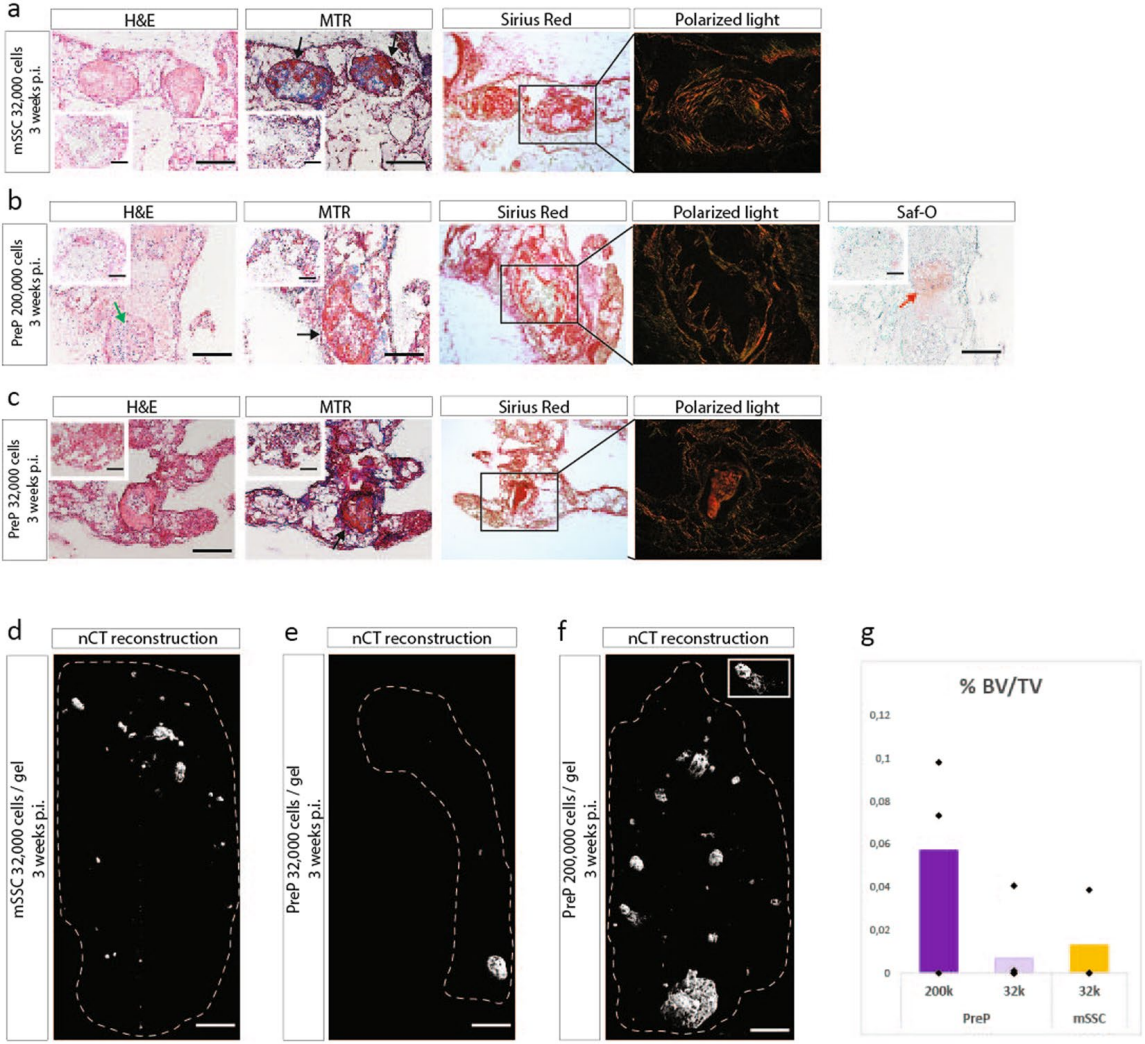
CD45^−^TER119^−^CD51^+^CD105^−^CD90.2^−^CD200^−^ PreP and CD45^−^TER119^−^CD51^+^CD105^−^CD90.2^−^CD200^+^ mSSC form small bone ossicles *in vivo* when encapsulated in alginate, an anchorage independent matrix. (**a**) mSSC develop into small immature bone ossicles after three weeks *in vivo*. (**b**) PreP at 200,000 cells per gel develop into small bone organoids, in which a proper long bone shape can be distinguished. (**c**) PreP 32,000 cells per gel are not able to form bone, only one replicate showed a small piece bone tissue in the hydrogel. Mature osteoid matrix (black arrows), cartilage tissue (red arrows), bone marrow sites (green arrows). (**d**–**f**) Reconstructed images of nCT of scanned explants: (**d**) mSSC, (**e**) PreP 32,000 cells per gel, (**f**) 200,000 cells per gel. Dashed line represents the border of the explant. (**g**) Quantification of bone volume over tissue volume (BV/TV), PreP and mSSC in alginate. Scale bars = 200 µm and 500 µm on the insets (**a–c**), 500 µm in (**d,e**) and 250 µm in (**f**). n = 3 for mSSC implants, n = 5 for PreP implants for *in vivo* implantation, n = 3 for bone tissue quantification.

When the mSSC and PreP were implanted in alginate, nCT after three weeks showed small islets of mineralized tissue distributed across the unabsorbed hydrogel in all conditions (Fig. 4d,e). Histological analysis showed that PreP implanted at 200,000 cells per gel showed several bone islets, in which growth plate-like Safranin-O positive structures could be observed (Fig. 4b). Ki67 staining detected proliferation in columnar chondrocytes, which matured to hypertrophy as shown by positive ColX staining (see Supplementary Fig. S5). Mason’s Trichrome staining showed osteoid matrix produced by osteoblasts, and thick organized collagen fibres could be observed. Bone marrow was also present.

In alginate, explants of mSSC and PreP at a concentration of 32,000 cells per gel generated very few bone islets (Fig. 4d,e). These islets comprised of few hypertrophic chondrocytes, but no columnar proliferative chondrocytes (see Supplementary Fig. S5). Although Mason’s Trichrome staining showed matured osteoid matrix, the organization of collagen fibres was less striking as compared to the implantations performed in collagen gel. Also, sporadic bone marrow was detected (Fig. 4a,c). Quantification of bone volume over tissue volume revealed very little amount of bone formed (Fig. 4g). All cells and tested densities had less than 0,1% bone tissue, in stark contrast with the bone tissue formed in the collagen hydrogels.

An overview of these results can be found in Supplementary Table S6. We showed that both mSSC and PreP sorted out of limb embryonic cartilage could form matured spherical bone ossicles, even at low cell densities in collagen gel. In alginate, small long bone organoids, dispersed throughout the hydrogel, were observed when PreP were implanted at high cell density. The formation of these ossicles by the PreP at a low cell density however was limited, since only 1 out of 8 explants showed skeletal tissue. The mSSC formed small spherical ossicles distributed across the alginate hydrogel, but here also the success rate was low. Overall, we showed the potential of the purified PreP and mSSC to form bone. However, this result is highly influenced by the presence of an appropriate biomechanical environment.

### FGF-2 supplementation enriches for the mSSC and PreP in expanded ECC *in vitro*, but does not preserve the biological bone forming potential *in vivo*

**Figure 5:**
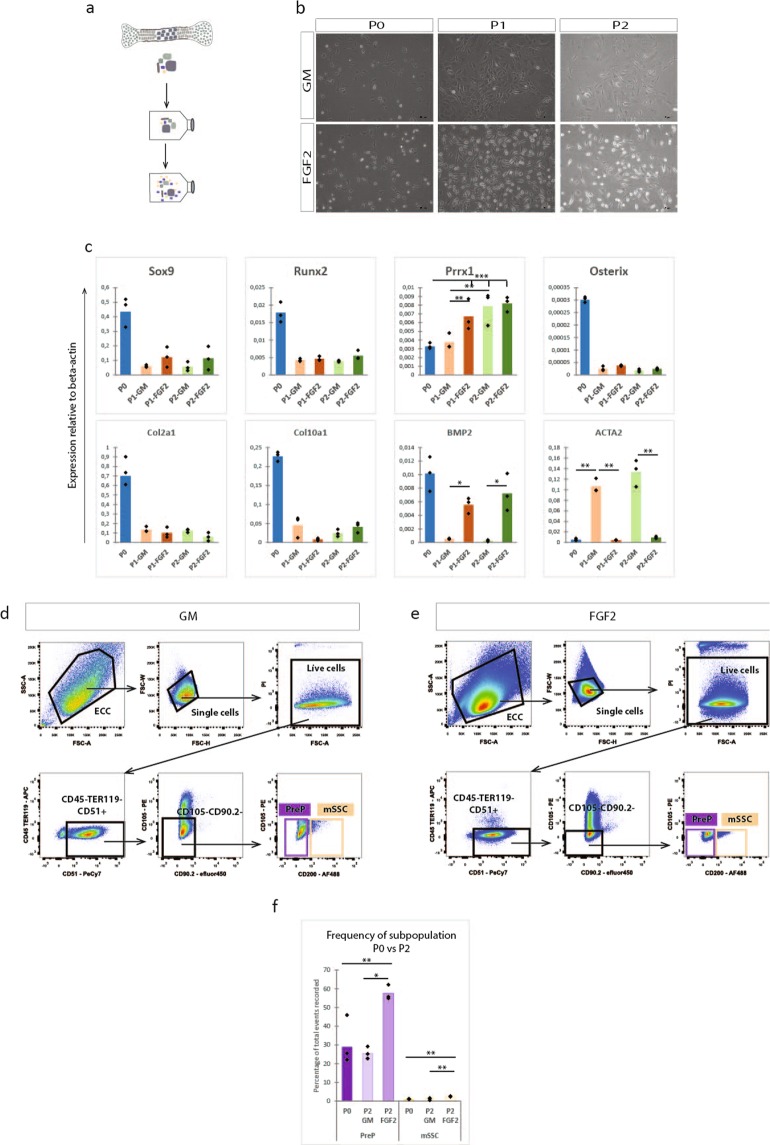
FGF2 enriches for CD45^−^TER119^−^CD51^+^CD105^−^CD90.2^−^CD200^−^ PreP and CD45^−^TER119^−^CD51^+^CD105^−^CD90.2^−^CD200^+^ mSSC in ECC *in vitro*. (**a**) Schematic overview of experiment. ECC were cultured up until passage two on a gelatin coated surface in standard growth medium (GM) or in standard growth medium supplemented with FGF2. (**b**) Bright field images of the ECC in culture at passage 0 (P0), passage 1 (P1) or passage 2 (P2) in GM or FGF2 medium. (**c**) Q-PCR analysis of mRNA transcript levels of endochondral markers (Sox9, Runx2, Osterix, Col2a1, Col10a1, BMP2), limb mesoderm (Prrx1) and fibroblastic contamination (ACTA2)). (**d**,**e**) Flow cytometry pseudocolour plots of P2-ECC, cultured in standard medium (**d**) or FGF2 medium (**e**) with the gating strategy for the presence of CD45^−^TER119^−^CD51^+^CD105^−^CD90.2^−^CD200^−^ PreP and CD45^−^TER119^−^CD51^+^CD105^−^CD90.2^−^CD200^+^ mSSC. (**f**) Graphical representation of the percentages of PreP and mSSC at passage 0 or passage 2 (standard or FGF2 expansion of ECC), relative to total events recorded. Statistical analysis for significance was performed with 2-way repeated measures ANOVA (**c**) and one way ANOVA (**f**) with Bonferroni post-hoc correction. n = 3 (**c**), n = 4 (**d**–**f**) *p < 0.05, **p < 0.01, ***p < 0.001.

Next, we tested a possibility to expand the embryonic cartilage cells prior to stem cell purification and implantation in order to obtain more cells for implantation. Plating of ECC in standard culture conditions (high glucose and 10% fetal bovine serum) induced dedifferentiation of the ECC towards a spindle shaped appearance. Expression of genes involved in endochondral ossification decreased rapidly and was undetectable already after one passage. Prior work^14^ showed that expanding and priming of murine periosteal progenitor cells with fibroblast growth factor 2 (FGF2) led to bone formation through the endochondral pathway both ectopically and in a large bone defect. This effect was mediated primarily by the increased expression of bone morphogenic protein 2 (BMP2). Thus, we investigated the addition of FGF2 to the culture media to prevent the dedifferentiation of the ECC *in vitro*. The ECC were expanded for two passages on a gelatin-coated surface and in standard growth medium or in the same medium supplemented with FGF2 (Fig. 5a). Gene expression analysis showed a decreased expression of Sox9, Runx2, Osterix, Col2a1 and Col10a1, without any significant differences between standard and FGF2 supplemented medium (Fig. 5c). Interestingly, an increase in Prrx1 could be seen in both culture media. While the osteochondrogenic differentiation was reduced, dedifferentiation of the ECC in FGF2 supplemented media was significantly less, as the increase in expression of fibroblastic marker ACTA2 was limited compared to the standard medium expansion. In FGF2 supplemented medium, the expression of BMP2 in the ECC could be sustained throughout the expansion, whereas the expression was almost completely lost in the standard conditions.

We compared the amount of mSSC and PreP in the ECC, expanded in standard (Fig. 5d) or FGF2 supplemented medium (Fig. 5e). In standard growth medium, no difference was observed in the amount of PreP or mSSC compared to P0-ECC. However, when ECC were expanded in FGF2 supplemented media, we detected a significant cell number increase in both populations, having 2.5 times more mSSC and double the amount of PreP (Fig. 5f). Thus, the FGF2 supplementation enriched ECC expanded *in vitro* for mSSC and PreP as defined by the CD markers.

**Figure 6:**
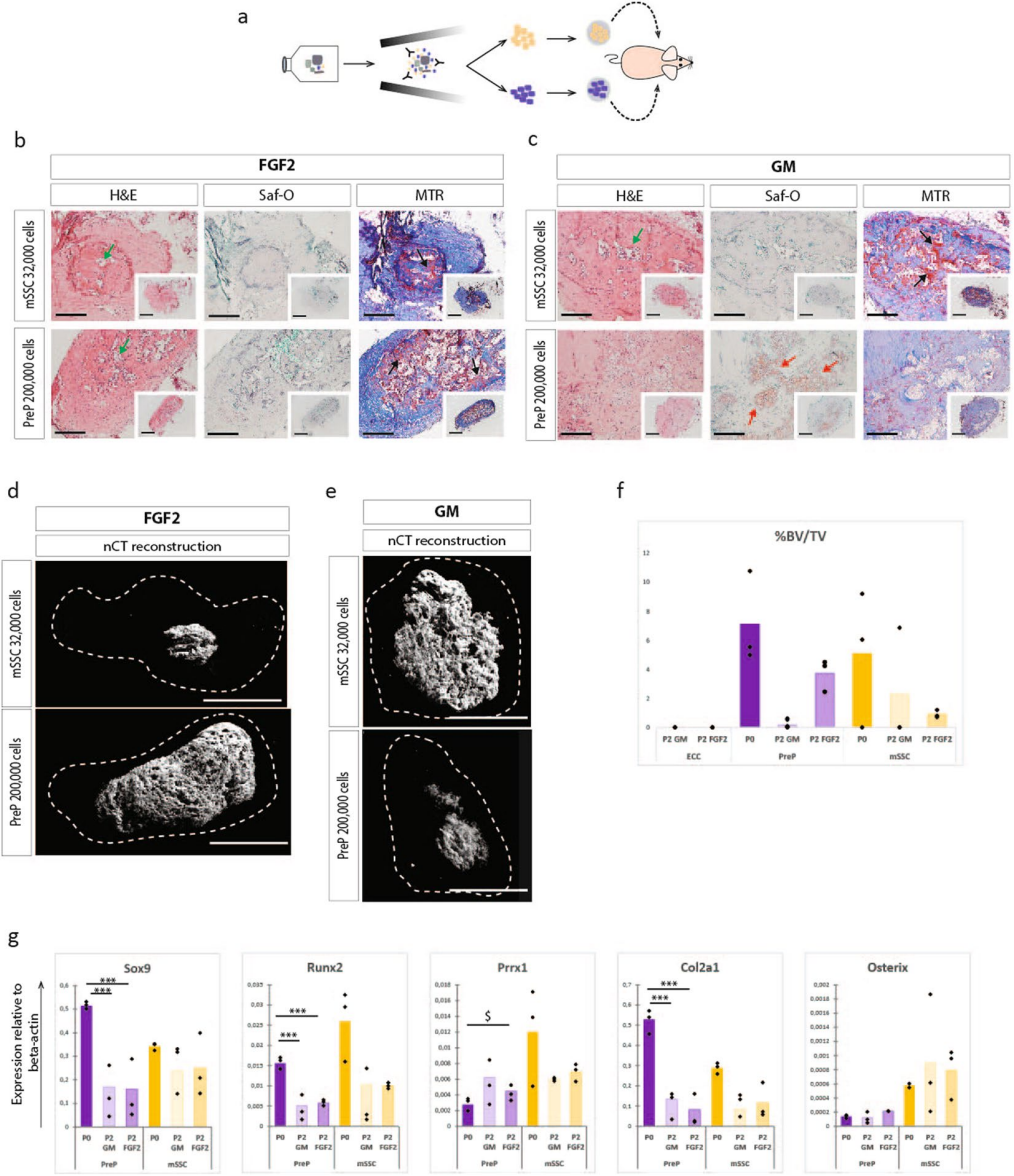
FGF2, despite stem cell enrichment, does not support all hallmarks of endochondral differentiation of mSSC and PreP fractioned from P2-ECC. (**a**) Schematic overview of experiment. ECC were cultured up until passage two in standard or FGF2 medium, followed by cell sorting of P2-mSSC and P2-PreP. These cells were encapsulated in collagen I hydrogel and implanted ectopically. (**b,c**) Histological analysis of explants of P2-mSSC and P2-PreP fractioned from ECC expanded in FGF2 medium (**b**) or standard medium (**c**), harvested 3 weeks p.i. Mature osteoid matrix (black arrows), cartilage tissue (red arrows), bone marrow sites (green arrows). (**d**,**e**) Reconstructed images from nCT scanned explants of P2-mSSC and P2-PreP from ECC expanded in FGF2 medium (**d**) or standard medium (**e**). Dashed line represents the border of the explant. (**f**) Quantification of bone volume over tissue volume (%BV/TV), comparing P2 ECC to PreP and mSSC at P0 and at P2. (**g**) Q-PCR analysis of mRNA transcript levels of endochondral markers of mSSC and PreP sorted from passage 0 ECC and from ECC expanded in standard medium or FGF2 medium. Scale bars = 200 µm and 500 µm in the inset in (**b,c**) 100 µm in (**d,e**) Statistical analysis for significance was performed by one way ANOVA with Bonferroni post-hoc correction. n = 3, *p < 0.05, **p < 0.01, ***p < 0.001, ^$^p = 0.05.

The penultimate test for differentiation potential is however an *in vivo* assay. Thus, ECC were expanded on gelatine in standard or FGF2 supplemented medium for two passages. PreP and mSSC from both culture conditions were sorted by FACS, encapsulated in collagen hydrogel and implanted ectopically in nude mice (Fig. 6a). The stem cells were implanted at a concentration of 32,000 cells and the progenitors at a concentration of 200,000 cells per gel. Explants were retrieved after three weeks. Macroscopic evaluation revealed a 50% reduction in size of explants from P2-mSSC and P2-PreP from expanded ECC in FGF2 medium compared to the explants of P0-mSSC and P0-PreP. Reconstructed nCT images of the explants (Fig. 6d) showed only mineralized tissue in the centre of the explants. Subsequent histological analysis (Fig. 6b) showed in P2-mSSC and P2-PreP (FGF2) explants fibrotic tissue with matured bone tissue in the centre, partially comprised of osteoid matrix (shown by positive red Mason Trichrome stain) and with sites of bone marrow formation. No cartilage matrix was detected.

We also implanted P2-mSSC and P2-PreP sorted from ECC expanded in standard growth medium as our negative control. These explants were also 50% smaller. Reconstructed nCT images showed similar structures of mineralized tissue in the centre (Fig. 6e). Histological analysis showed no bone tissue except in one P2-mSSC explant. One P2-PreP explant showed Safranin-O positive hypertrophic cartilage, without any bone tissue (Fig. 6c). Quantification of bone showed a decrease in bone volume over tissue volume for the progenitors purified from P2-ECC compared to P0-progenitors (Fig. 6g). These results are summarized in Supplementary Table S7. Subsequent gene expression analysis of osteochondrogenic markers in P2-mSSC and P2-PreP (standard growth medium and FGF2) showed a significant decreased expression for Sox9, Runx2 and Col2a1 when compared with P0-mSSC and P0-PreP. Prrx1 expression was maintained in P2-PreP (standard) or even slightly increased in P2-PreP (FGF2), but decreased significantly in P2-mSSC (standard and FGF2). Osterix expression was unchanged in P2-mSSC, although subjected to high variation (Fig. 6g).

Thus, even though FGF2 expansion enriched for stem cells and progenitors in embryonic limb cartilage, a major reduction in bone formation capacity was observed. Subsequent gene expression analysis revealed a decrease in all genes relevant to osteochondral ossification, implying the reduced osteochondral potential of P2-mSSC and P2-PreP.

## Discussion

Developmental engineering aims to regenerate or repair damaged tissues by recapitulating the programs and processes taking place during development *in vivo*.

We focused on cell populations derived from 14.5 dpc limb embryonic cartilage as they contain cells representing all the differentiation steps of chondrogenesis. Earlier stages of embryonic limb cartilage were not used for several reasons. Firstly, the isolation of the limb cartilage was performed manually under a dissection microscope and the earlier developmental stages were not amenable to this type of manipulation because of high risk of contamination by surrounding tissue. Additionally, since the anlagen are smaller, the amount of released cells would be lower, increasing the animal load. Also, it has been reported that embryonic cartilage cells from less mature embryonic limbs, when implanted ectopically, were not able to form bone efficiently^15^. This supports the developmental engineering concept that one must create a tissue intermediate sufficiently matured to contain all the information to drive the tissue formation in a postnatal environment in a robust and semi-autonomous manner^16^.

We encapsulated the embryonic cartilage cells in either collagen or alginate hydrogel for implantation *in vivo*. It must be noted however that encapsulation of the ECC in collagen or alginate hydrogel does not accurately recapitulate the developmental context. During development, these cells experience more cell-cell interactions and less cell-matrix (in this case collagen II). For the collagen hydrogel, the shape of the implant can be adjusted depending on the mould in which the gel is allowed to set, increasing its versatility to use in different defect sizes or shapes. Alginate, a natural anionic polysaccharide polymer obtained from brown seaweed, is been studied extensively in biomedical engineering due to its biocompatibility, low toxicity, relative low cost and mild gelation methods^17^. Alginate has several advantages over non-injectable 3D scaffolds when used for bone regeneration. It can be introduced into the body in a minimally invasive manner; it can fill irregular shaped defects and can be used as delivery system for slow release of bone inducing factors. The disadvantages are insufficient mechanical properties to allow load bearing in the initial stages of regeneration and inability to degrade under physiological conditions. Nonetheless, alginate is frequently used for cartilage generation and anchorage independent chondrocyte cultures. Chondrogenic potential of stem cells seems linked to the morphology of the encapsulated cells (preferably preservation of the round cell shape) and alginate promotes this rounded shape and thus the cellular differentiation and maturation process^18,19^. We have shown that the embryonic cartilage cells form cartilage and bone after three weeks *in vivo* when encapsulated in collagen I hydrogel. Thus, the cells were able to maintain their developmental fidelity, suggesting that the bone formation is a cell intrinsic process. This has been demonstrated by previous work in our group^20^. However, when the cells were encapsulated in alginate, bone formation was very limited. Thus, a scaffold that provides a niche to allow initial cell attachment has a great influence on the cells potential and in which direction their differentiation goes. Furthermore, the alginate might keep the cells in a more chondrogenic state, thereby hindering further development into bone. Additionally, it must be noted that with alginate the invasion of blood vessels and recruitment of osteoblasts is limited. Nonetheless, we detected small areas of osteoid positive matrix in the hypertrophic cartilage tissue islands in the alginate gel. In the cell-tracing studies, we detected eGFP^+^Osterix^+^ donor cells beyond the cartilage-bone perimeter, in the osseous tissue. These results combined may suggest differentiation of donor cells to osteoblasts. Contamination of the cell preparation with osteoblastic precursors could not be detected by qPCR, but cannot be excluded. Therefore, donor cells contributing to the osseous tissue could be osteoblastic precursors, or hypertrophic chondrocytes undergoing transdifferentiation. Recently, Yang, *et al*.^21^ was able to tag Col10a1 expressing hypertrophic chondrocytes during mouse development and adulthood and track their fate. They showed survival of these cells during the cartilage-to-bone transition and differentiation into osteoblasts in foetal and postnatal endochondral bone, which could persevere into adulthood. Studies are now being undertaken to further investigate the molecular pathways underlying this transdifferentiation phenomenon^22,23^.

Recent lineage tracing studies in mice investigated the existence of a mouse skeletal stem cell which contributes to the skeletal tissues during development, homeostasis and regeneration. However in these studies, the focus was on genes and proteins expressed intracellularly, making it difficult to study these cell populations outside of their natural environment and in a tissue engineering context. The identification of the mouse skeletal stem cell and its downstream progenitors by Chan, *et al*.^13^, and later the publication of the detailed protocol^24^ enabled studies of the skeletal stem cell hierarchy, whether in long bone fracture healing studies^25^, disease models such as diabetes mellitus^26^ or for tissue engineering purposes for healing large bone defects (our study).

Embryonic cartilage cells obtained from femur anlagen of 14,5dpc mouse embryos contain about 1% of mSSC. This is significantly more than 0.1% reported in previous studies^13^. The most likely reason for this discrepancy is that the previous report used young adult (P6) mice, thus much older than our material. These findings support the general notion that the amount of stem cells decreases with age of the individual. It would be interesting to investigate whether there is a higher percentage of stem cells earlier in development, at the onset of limb development at 10.5dpc, when the cells already express Gremlin, a proposed stem cell marker^9^.

Besides endochondral relevant genes, we measured the expression of Meis1 and Hox genes, more specifically the analogs 9–11. These genes show a proximal-distal expression pattern, in which Meis1 is expressed most proximally, Hoxa9 and Hoxa10 is expressed in stylopod and Hoxa11 in the zeugopod. We observed an increased expression of Hoxa10 in mSSC and PreP. Loss of Hox10 paralogous genes results in severe stylopod mispatterning. Our result that Hoxa10 is slightly upregulated in mSSC isolated from stylopod limb cartilage is therefore an interesting finding. However, the expression of these genes is not uniform and dependent on the tissue type. Additional, in literature, an involvement of Hoxa10 in bone formation has been reported. The gene responds to BMP2 stimulation, and thereby contributes to osteogenic lineage specification through activation of Runx2 and directly regulates osteoblastic genes such as alkaline phosphatase, osteocalcin and bone sialoprotein genes^27^.

In our study, we designed a protocol to investigate the potential of the mSSC and the PreP to form bone when encapsulated in a hydrogel in our established *in vivo* bone tissue engineering assay.

While preparing the manuscript, a detailed protocol regarding the isolation and functional assessment of the skeletal stem cell was published^24^ making a comparison between these two procedures possible (Table 1). We used collagen I or alginate as a carrier hydrogel for the cells, instead of Matrigel. The latter contains structural components such as laminin, collagen, heparan sulphate and growth factors such as TFG-β and EGF, which can influence the differentiation of the encapsulated cells. Since its exact composition varies from a lot to a lot, the tissue formation process cannot be well controlled, making it less suitable for tissue engineering applications and not suitable from the regulatory point of view.

**Table 1:** Overview of the protocol published in literature, and our optimised tissue engineering based protocol.

Different aspects of the *in vivo* functional assessment	Chan, *et al*.^13,24^	This study
Hydrogel	Matrigel	Collagen I or alginate
Number of cells implanted/gel	2,500–5,000 cells in 2uL	32,000 or 200,000 cells in 50 uL
# cells/uL hydrogel	1,250–2,500 cells/uL	640–4,000 cells/uL
Location of implantation	Kidney capsule	Subcutaneously
*In vivo* tissue formation period	4–6 weeks	Max 3 weeks

We were able to sort a maximum of 32,000 mSSC from embryonic anlage cells pooled from 40–50 embryos. These cells were encapsulated in 50 uL hydrogel, resulting in a very low cell seeding density of 640 cells per uL of hydrogel. In standard tissue engineering protocols, 1 million cells or more are encapsulated in hydrogel in a volume ranging from 50 to 100 uL. Our implantation site was located subcutaneously in the flank region right behind the shoulder of immunocompromised NRMI nu/nu mice, as opposed to the less accessible kidney capsule, making the experimental protocol easier, faster and with less stress to the animal. In collagen, the fractioned mSSC and PreP were sufficiently potent to form complete mature bone ossicles at low seeding densities already after three weeks *in vivo*. Calculated bone volume showed a trend for more bone when implanting mSSC or Prep in comparison to ECC suggesting that one can reduce cell number and that the markers and subsequent purification have a beneficial effect on the potential of the implanted cell population to form bone. This effect was also observed in the alginate explants of the sorted cells, but the effect here was limited. No bone was observed when implanting ECC, but some bone was formed in mSSC and PreP explants. However, this amount was significantly lower compared the bone formed in collagen gel. Differences in the response of cells to alginate or collagen are described above. Yet, implantation of PreP in alginate gave rise to an interesting result. In those implants, we detected not only mineralized tissue islands but also structures resembling long bones. In the centre of these tissue structures, proliferative chondrocytes were located maturing to hypertrophy in a longitudinal direction, eventually followed by bone formation at the epiphysis. It is tempting to speculate that the PreP retain positional information, allowing them to form tissue along the correct axis, even in the absence of mechanical stimulus. Since the PreP at a lower cell seeding density were not able to make these structures, a threshold for minimal cell density and signals to create these tissues exists, suggesting a community effect as a prerequisite for proper tissue formation^28^.

One of the most severe limitations when working with primary cells is their availability. Therefore, a cell expansion step is typically used to bypass this limitation and increase the number of cells for downstream applications. A challenge when expanding primary cells is to find the conditions to preserve their identity and, most importantly, their *in vivo* potential, while increasing their number. Since we needed embryos from three pooled litters (approximately 40–50 embryos in total) to isolate sufficient number of stem cells and progenitors, we sought to develop an *in vitro* expansion protocol for these embryonic cartilage cells. One of the ligands typically used in such applications is FGF2^29,30^. We were able to expand the ECC almost 10-fold over two passages, leading to sufficient cell numbers for isolation of mSSC and PreP. The application of FGF2 did help in the maintenance of BMP2 expression and the inhibition of ACTA2, a fibroblast marker as compared to standard growth medium. Subsequent flow cytometry analysis showed an enrichment for mSSC and PreP after two passages of expanding ECC in the presence of FGF2. However, when introducing the fractioned cells in the ectopic assay, the *in vivo* bone formation was significantly reduced and large amounts of fibrotic tissues was found instead. Additionally, no significant difference in bone tissue was found between progenitors isolated from ECC expanded in FGF2 or GM. Subsequent gene expression analysis of P2-mSSC and P2-PreP showed a significant reduction in the expression levels of genes driving osteochondral ossification compared to P0-mSSC and P0-PreP. Even though the FGF2 supplementation led to an increase in the cell number of the investigated progenitor populations as defined by the CD marker set, it did not preserve their capability to form bone tissue according to the endochondral ossification pathway. Therefore, we concluded that in our expansion protocol, the reported stem cell markers may not be useful for prospective cell expansion and selection, and more importantly for successful *in vivo* bone formation. It must be noted that this study is performed on embryonic cells, and that young/adult cells could give different results. It is known that the chromatin of embryonic cells is less compact and more permissive to change than cells that are more adult. In embryonic cells, promoters of many developmental regulator genes share an unusual bivalent chromatin pattern, which keep these genes in a poised conformation, ready for expression or silencing in response to cues^31^. Adult stem cells dissected from their specific niches had more time to fix their epigenetic signature determining their cellular identity. Similar experiments using adult cells could give more insights into these aspects.

## Methods

### Mice

All animal experiments were conducted according to the European and Belgian legislation and with approval of the Animal Ethics Committee of the faculty of Biomedical Sciences of the KU Leuven (project license 221/2015). SWISS, NMRI nu/nu mice (Janvier labs) and B6.Cg-Tg(ACTb-eGFP) mice were used in these studies. For timed matings, male and female SWISS mice were placed together and left overnight. The next day (0.5 dpc) the success of the mating was assessed by the presence of a vaginal plug. At 14.5 dpc pregnant females were sacrificed by cervical dislocation.

### Cell isolation and culture

Embryos were harvested, staged, and the femurs were dissected and digested with collagenase II (Thermo Scientific) for 40 min at a concentration of 24 mg/ml in standard growth medium (DMEM High glucose supplemented with 10% FBS, 1% antibiotics/antimyotics, 1% sodium pyruvate – Thermo Scientific) under continuous rotation. After digestion, the embryonic cartilage cells were passed through a 70 µm cell strainer to obtain a single cell suspension. Cells were expanded *in vitro* by seeding them on pre-coated gelatin surface (0,1% - Millipore) at 20,000 cells/cm^2^ and cultured in a humidified incubator at 37 °C with 5% CO_2_ in standard medium with or without supplementation with 10 ng/mL FGF2 (Peprotech). Upon reaching 90% confluence, the cells were passaged twice using the same media conditions.

### Flow cytometric analysis and fluorescent activated cell sorting

Single cells suspended in 2% FBS in PBS (Lonza) were stained with fluorochrome-conjugated antibodies against CD45, TER119, CD51, CD200, CD105 and CD90.2 (Ebioscience – Thermo Scientific). All antibodies were used at their optimal concentrations as determined by titration. Three controls were used: unstained cells, single stained compensation controls (UltraComp eBeads – Thermo Scientific) and fluorescence-minus-one (FMO) controls. An FMO control contains all the tested antibodies in the panel, minus one of them. This gives a strong negative control to work with since it allows to take into account how the other stains in the panel influence the left out parameter. These FMOs were used as gating controls to accurately discriminate positive vs negative signals. The step-by-step gating strategy is described in Fig. 2. Flow cytometric analysis was performed on a BD Canto II HTS equipped with a blue, red and violet laser. Cell fractionation was performed on the BDFACS Aria III (with a blue, red and near UV laser), and the stem cells and progenitors were sorted in 20% FBS in PBS. Data analysis was performed using FlowJo software (Tree Star). Frequency of cell populations is shown as frequency to total number of events recorded and data is displayed as pseudocolour plots.

### Ectopic *in vivo* bone formation assay

Single cells (ECC, mSSC, PreP) were encapsulated in two different hydrogels: collagen or alginate, at a volume of 150 uL or 50 uL. Single cells were pelleted down, suspended in 5 mg/mL collagen gel I (Corning) prepared according to the manufacturer’s protocol and allowed to gelate in moulds for 30–40 min at 37 °C. Similarly, pelleted cells were suspended in 1,5% alginate (Sigma) and chemically crosslinked with 102 mM CaCl_2_ (Sigma). Either of the gels were subcutaneously implanted behind the shoulders of NMRI nu/nu mice, anaesthetized with ketamine-xylazine (80 mg/kg b.w.t. ketamine and 5 mg/kg b.w.t xylazine - IP). After implantation, the mice received analgesics (Buprenorphin 0,05–0,1 mg/kg – IP). After one, two or three weeks, gels were retrieved for *ex vivo* nano computed tomography (nCT) and histological analysis.

### Cell tracing experiments

For cell tracing experiments, ECC were derived from ACTb-eGFP mice and encapsulated in collagen I gel as described above. Implants were retrieved after one, two or three weeks. Retrieved explants were fixed in 4% paraformaldehyde overnight at 4 °C, washed with PBS and decalcified in 0.5 M EDTA/PBS solution for 14 changes (of 24 h) prior to 30% sucrose cryoprotection and embedding in OCT (Optimal Cutting Temperature embedding material - VWR). Samples were sectioned on a cryostat (Microm HM560) at 6 µm and stored at −80 °C until staining. Dual immunofluorescence staining for eGFP and Sox9, Runx2 or Osterix (Txf) was performed by combining an anti-eGFP (Abcam: ab139700) and anti-Txf (Sox9, Runx2 or Osx; Abcam: ab185966, ab192256 and ab22552 respectively) (1/1000 in blocking buffer: 5% goat serum in TNB) primary antibody. After 1.5 h, secondary antibodies Dylight 488 (Abcam: ab96951) and Alexa Fluor 546 (Life Technologies) (1/200 and 1/500 in blocking buffer respectively) were incubated for 1 h. The samples were counterstained with Hoechst 33342 (1/200 in PBS) (stock solution 10 mg/mL - Sigma) for 5 min and mounted with Fluoroshield mounting medium (Sigma). The mounted samples were imaged using a fluorescence microscope (Olympus BX63).

### Quantification of new bone tissue by *ex vivo* nanofocus computed tomography

Retrieved explants were fixed in 4% paraformaldehyde overnight at 4 °C, washed with PBS and stained with 20% Hexabrix in PBS overnight at room temperature and imaged using a Phoenix NanoTom M (GE Measurement and Control Solutions). To generate the X-rays, a diamond-coated tungsten target was applied. The system was operated at a voltage of 60 kV and a current of 190 µA, and a 0,1 mm aluminium filter was used to reduce beam hardening during the acquisition. The exposure time was 500 ms and 2400 images were acquired over 360° using the fast scan mode (frame averaging = 1 and image skip = 0). The explants were examined at 3.02 µm isotropic voxel size. During reconstruction (Datos |x, GE Measurements and Control Solutions), a beam hardening correction of 8 was applied. Region of interest selection, thresholding and bone volume over tissue volume (BV/TV, %) calculations were determined using CTan software (Bruker).

### Histological evaluation

Explants were decalcified in 0.5 M EDTA/PBS solution for 14 changes (of 24 h) prior to dehydration, embedding in paraffin wax and sectioning at 5 µm. Slides were stained with Haematoxylin & Eosin, Safranin-O and Fast Green, Mason’s Trichrome and Picrosirius Red. Images were taken with an Olympus DP73 microscope. Polarized light microscopy to visualize collagen fibers after Picrosirius Red staining was performed with a Leica DMR microscope and SPOT imaging software.

### Immunohistochemistry staining

Proliferation and hypertrophy were detected with Ki67 and ColX immunohistochemistry staining respectively. Briefly, slides were dewaxed, dehydrated and hydrated before blocking cellular peroxidase activity with H_2_O_2_. For Ki67, an antigen retrieval step (10 m Sodium citrate pH6 + 0,05% Tween-20) was performed. Primary antibodies targeting Ki67 (Abcam ab16667) or ColX (Cosmo Bio LS-LB-0092) were incubated overnight in blocking buffer (TBS with 5% goat serum). The next day, the biotynilated secondary antibody (Abcam ab6720) was incubated for 30 min. An ABC kit (Vectastain PK4000) was added to amplify the biotin-streptavidin reaction and provide the detection by HRP and the DAB substrate (DAKO K346711). Slides were counterstained with Haematoxylin and mounted with Pertex.

### RNA extraction and quantitative reverse transcriptase polymerase chain reaction analysis

RNA from primary, cultured or sorted cells was extracted using the RNeasy Mini Kit (Qiagen) or RNeasy Micro Kit (Qiagen) and converted to cDNA with the Revert Aid H Minus First strand cDNA synthesis kit (Thermo Scientific) according to the manufacturer’s protocols. Quantification of gene expression was carried out using SYBR Select (Master mix - Applied Biosystems) based qPCR reaction on the StepOne Plus System (Applied Biosystems). Expression levels were analysed using the 2^−ΔCt^ method and normalized for the expression of the reference gene β-actin. Specific forward and reverse oligonucleotide primers were used, and are listed in Supplementary Table S8.

### Statistical analysis

Biological independent replicates for each experiment are indicated in the respective figure legends. Data was analysed in SPSS (v22, IBM Analytics). Details of the statistical tests performed for each experiment can be found in the representative figure legends. Graphs show raw data as individual data points, overlaid with a bar indicating the mean. We present a typical histological section from each of the experiments.

## Supplementary information

Supplementary information

## References

[1] Arrington, E. D., Smith, W. J., Chambers, H. G., Bucknell, A. L. & Davino, N. A. Complications of iliac crest bone graft harvesting. *Clinical orthopaedics and related research*, 300–309 (1996). 10.1097/00003086-199608000-00037 8769465

[2] Banwart JC, Asher MA, Hassanein RS (1995). Iliac crest bone graft harvest donor site morbidity. A statistical evaluation. Spine.

[3] Greenwald AS (2001). Bone-graft substitutes: facts, fictions, and applications. The Journal of bone and joint surgery. American volume.

[4] Kozhemyakina E, Lassar AB, Zelzer E (2015). A pathway to bone: signaling molecules and transcription factors involved in chondrocyte development and maturation. Development (Cambridge, England).

[5] Roberts SJ, van Gastel N, Carmeliet G, Luyten FP (2015). Uncovering the periosteum for skeletal regeneration: the stem cell that lies beneath. Bone.

[6] Colnot C, Lu C, Hu D, Helms JA (2004). Distinguishing the contributions of the perichondrium, cartilage, and vascular endothelium to skeletal development. Dev Biol.

[7] Bolander J (2017). Healing of a Large Long-Bone Defect through Serum-Free *In Vitro* Priming of Human Periosteum-Derived Cells. Stem Cell Reports.

[8] Duchamp de Lageneste O (2018). Periosteum contains skeletal stem cells with high bone regenerative potential controlled by Periostin. Nat Commun.

[9] Worthley DL (2015). Gremlin 1 identifies a skeletal stem cell with bone, cartilage, and reticular stromal potential. Cell.

[10] Zhou BO, Yue R, Murphy MM, Peyer JG, Morrison SJ (2014). Leptin-receptor-expressing mesenchymal stromal cells represent the main source of bone formed by adult bone marrow. Cell Stem Cell.

[11] Shi Y (2017). Gli1 identifies osteogenic progenitors for bone formation and fracture repair. Nat Commun.

[12] Mendez-Ferrer S (2010). Mesenchymal and haematopoietic stem cells form a unique bone marrow niche. Nature.

[13] Chan CK (2015). Identification and specification of the mouse skeletal stem cell. Cell.

[14] van Gastel N (2014). Expansion of murine periosteal progenitor cells with fibroblast growth factor 2 reveals an intrinsic endochondral ossification program mediated by bone morphogenetic protein 2. Stem cells (Dayton, Ohio).

[15] Fernando, W. A. *et al*. Limb derived cells as a paradigm for engineering self-assembling skeletal tissues. *J Tissue Eng Regen Med*, 10.1002/term.2498 (2017). 10.1002/term.2498 28603948

[16] Lenas P, Moos M, Luyten FP (2009). Developmental engineering: a new paradigm for the design and manufacturing of cell-based products. Part I: from three-dimensional cell growth to biomimetics of *in vivo* development. Tissue engineering. Part B, Reviews.

[17] Lee KY, Mooney DJ (2012). Alginate: properties and biomedical applications. Progress in polymer science.

[18] Dashtdar H (2011). A preliminary study comparing the use of allogenic chondrogenic pre-differentiated and undifferentiated mesenchymal stem cells for the repair of full thickness articular cartilage defects in rabbits. Journal of orthopaedic research: official publication of the Orthopaedic Research Society.

[19] Tuan RS, Boland G, Tuli R (2003). Adult mesenchymal stem cells and cell-based tissue engineering. Arthritis Res Ther.

[20] Fernando WA (2018). Limb derived cells as a paradigm for engineering self-assembling skeletal tissues. Journal of Tissue Engineering and Regenerative Medicine.

[21] Yang L, Tsang KY, Tang HC, Chan D, Cheah KS (2014). Hypertrophic chondrocytes can become osteoblasts and osteocytes in endochondral bone formation. Proceedings of the National Academy of Sciences of the United States of America.

[22] Wong SA (2018). Microenvironmental Regulation of Chondrocyte Plasticity in Endochondral Repair-A New Frontier for Developmental Engineering. Frontiers in bioengineering and biotechnology.

[23] Aghajanian P, Mohan S (2018). The art of building bone: emerging role of chondrocyte-to-osteoblast transdifferentiation in endochondral ossification. Bone Research.

[24] Gulati, G. S. *et al*. Isolation and functional assessment of mouse skeletal stem cell lineage. *Nature Protocols* **13**, 1294, 10.1038/nprot.2018.041, https://www.nature.com/articles/nprot.2018.041#supplementary-information (2018). 10.1038/nprot.2018.041 PMC6530903 29748647

[25] Marecic O (2015). Identification and characterization of an injury-induced skeletal progenitor. Proceedings of the National Academy of Sciences.

[26] Tevlin, R. *et al*. Pharmacological rescue of diabetic skeletal stem cell niches. *Sci Transl Med* **9**, 10.1126/scitranslmed.aag2809 (2017). 10.1126/scitranslmed.aag2809 PMC5725192 28077677

[27] Hassan MQ (2007). HOXA10 Controls Osteoblastogenesis by Directly Activating Bone Regulatory and Phenotypic Genes. Molecular and Cellular Biology.

[28] Gurdon JB, Tiller E, Roberts J, Kato K (1993). A community effect in muscle development. Current biology: CB.

[29] Eiselleova L (2009). A complex role for FGF-2 in self-renewal, survival, and adhesion of human embryonic stem cells. Stem cells (Dayton, Ohio).

[30] Lotz S (2013). Sustained levels of FGF2 maintain undifferentiated stem cell cultures with biweekly feeding. PloS one.

[31] Spivakov M, Fisher AG (2007). Epigenetic signatures of stem-cell identity. Nature Reviews Genetics.

