# Supplementary material for: Uncoupling of in-vitro identity of embryonic limb derived skeletal progenitors and their in-vivo bone forming potential
Source: Sci Rep. 2019 Apr 8;9:5782. doi: 10.1038/s41598-019-42259-x (PMC6453955; doi:10.1038/s41598-019-42259-x)
Supplement: Supplementary file 1 — Supplementary information [file 41598_2019_42259_MOESM1_ESM.docx]

**Uncoupling of *in-vitro ­*identity of embryonic limb derived skeletal progenitors and their *in-vivo* bone forming potential**

***Louca Verbeeck^1,2^, Liesbet Geris^1,4,5^, Przemko Tylzanowski^3,6, $^, Frank P. Luyten^1,2,3,$,*^***

*^1^Prometheus, Div of Skeletal Tissue Engineering, KU Leuven, Belgium*

*^2^Tissue Engineering laboratory, SBERC, KU Leuven, Belgium*

*^3^Development & Stem Cell Biology laboratory, SBERC, KU Leuven Belgium*

*^4^Biomechanics Research Unit, University de Liege, Belgium*

*^5^Biomechanics Section KU Leuven, Belgium*

*^6^Dept of Bioch. & Mol Biol., Medical University Lublin, Poland*

*^$^ Joint senior author*

*^*^ Correspondence:* [*frank.luyten@uzleuven.be*](mailto:frank.luyten@uzleuven.be)

**Supplemental information**


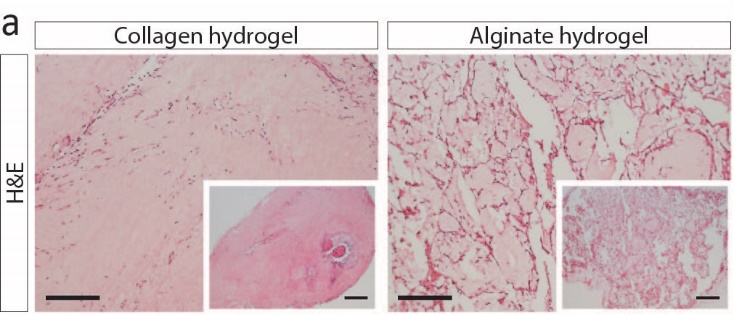


**Supplementary Figure S1: Negative controls for implantation** (**a**) H&E staining of explant of empty collagen hydrogel (left panel) and empty alginate hydrogel (right panel) after three weeks *in vivo.* Scale bars = 200µm and 500µm in the inset. n = 3 for each hydrogel.


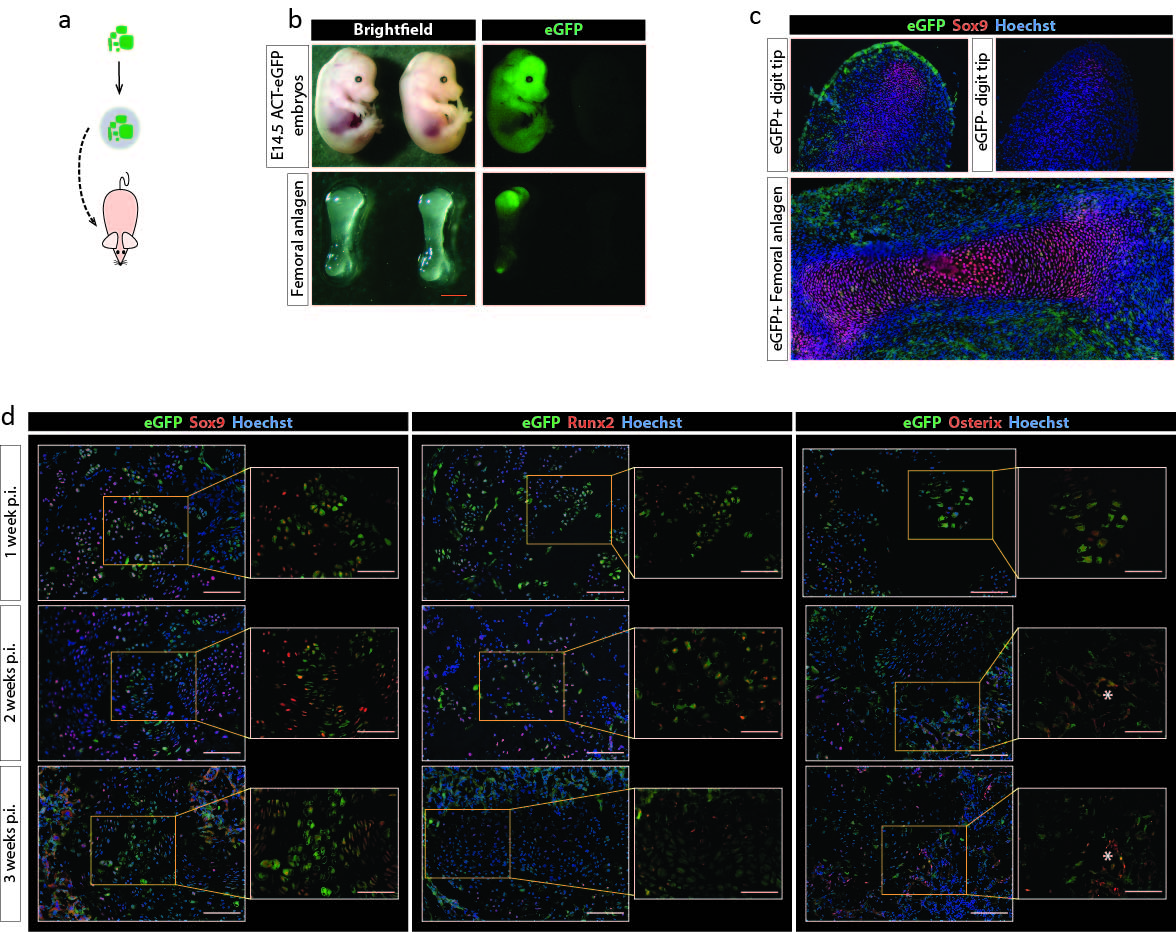


**Supplementary Figure S2: Donor cells differentiate into osteoblasts and contribute to the osseous tissues *in* *vivo*.** (**a**) Schematic overview of experiment. ECC from ACTb-eGFP embryos were encapsulated in collagen and implanted *in* *vivo.* (**b**) ACTb-eGFP embryos 14.5dpc and femoral anlagen images with bright field microscopy (left panel) and with fluorescence microscopy (right panel). (**c**) Sections of eGFP^+^ and eGPP^-^ embryonic digit tips and of eGFP^+^ femoral anlagen, simultaneously immunostained with antibodies against eGFP (green) and Sox9 (red). Cell nuclei are stained in blue (DAPI). (**d**) Dual immunofluorescent staining of explants retrieved after one, two or three weeks of eGFP with Sox9 (left panel), Runx2 (middle panel) or Osterix (right panel). After three weeks *in vivo*, Osterix expressing eGFP^+^ donor cells were observed in the formed osseous tissue in the explants (white asterix). Scale bars 200µm and 500µm in the inset in (**d**). n = 6 for each time point.


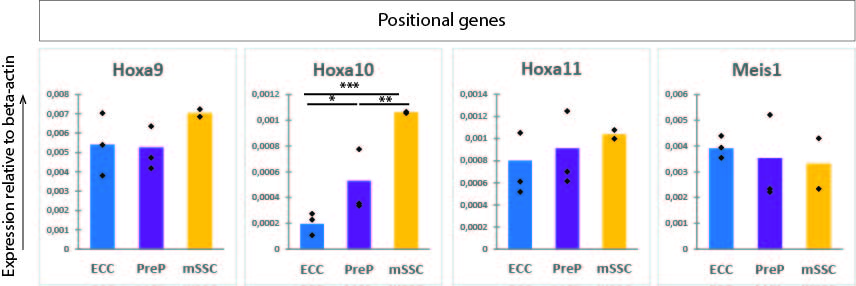


**Supplementary Figure S3: mSSC show an increased expression of Hoxa10, a gene involved in positional proximal-distal limb development.** Gene expression analysis of genes involved in positional information in limb development. Statistical analysis for significance was performed with 1 way ANOVA with Bonferroni post-hoc correction. n = 2/3, *p < 0.05, **p < 0.01, ***p < 0.001


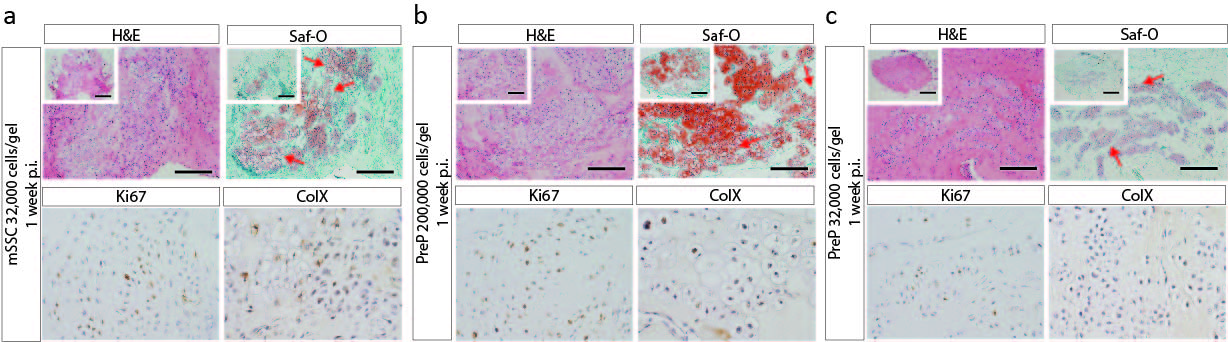


**Supplementary Figure S4: mSSC and PreP form bone via the endochondral program** (**a**), (**b**) and (**c**) show the presence of cartilage (red arrows) at one week p.i., together with immunohistochemistry for proliferative chondrocytes (Ki67) and hypertrophic chondrocytes (ColX), indicated by the brown staining. Scale bars = 200µm and 500µm on the insets. n = 3 for mSSC implants, n = 6 for PreP implants.

**
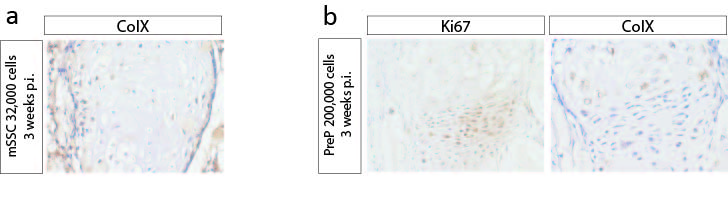
**

**Supplementary Figure S5: Longitudinal growth plate zonations are detected in PreP explants in alginate.** (**a**) and (**b**) immunohistochemistry for Ki67 and ColX, showing proliferative and hypertrophic chondrocytes in mSSC (**a**) and PreP (**b**) explants.

**Supplementary Table S6: Summary of results of implantations of P0-mSSC and P0-PreP in either collagen I or alginate hydrogel.** An explant containing bone and/or cartilage is seen as a positive outcome. Data is shown as positive results/total number of explants.

|  | mSSC | PreP | |
| --- | --- | --- | --- |
| *Cell density* | *32,000 cells* | *32,000 cells* | *200,000 cells* |
| Collagen I | 3/4 | 4/6 | 5/5 |
| Alginate | 1/4 | 1/8 | 3/5 |

**Supplementary Table S7: Summary of results of P2-mSSC and P2-PreP fractioned from ECC expanded in standard growth medium of FGF2.** An explant containing bone and/or cartilage is seen as a positive outcome. Data is shown as positive results/total number of explants.

|  | mSSC | PreP |
| --- | --- | --- |
| *Cell density* | *32,000 cells* | *200,000 cells* |
| Standard growth medium expanded | 1/3 | 1/5 |
| FGF2 expanded | 2/3 | 4/5 |

**Supplementary Table S8:** **Overview of used oligonucleotides for Q-PCR reaction.** Primers were designed by the Primer Design tool of NCBI.

| Gene | Forward primer | Reverse primer |
| --- | --- | --- |
| β-actin | CGGTTCCGATGCCCTGAGGCTCTT | CGTCACACTTCATGATGGAATTGA |
| Sox9 | GAAGAACGGACAAGCGGAGG | GGACCCTGAGATTGCCCAGA |
| Col2a1 | TCATCTTGCCGCATCTGTGTG | CCCTTTGGCCCTAATTTTCCACT |
| Prrx1 | GGAAGACACTGAAAAGCGCCAT | AGTTGACTGTTGGCACCTGG |
| Runx2 | TCCCCATCCATCCACTCCAC | GTTCTGAAGCACCTGCCTGG |
| Col10a1 | TCCCAGCACCAGAATCTATCTGA | TTATGCCTGTGGGCGTTTGG |
| Osterix | ACAAGAGTGAGCTGGCCTGA | AGTGAGCTTCTTCCTGGGTAGG |
| ACTA2 | CCATCTTTCATTGGGATGGAGTCA | ACCCCCTGACAGGACGTTG |
| BMP2 | AGATCTGTACCGCAGGCACT | GTTCCTCCACGGCTTCTTCG |
| Meis1 | AGAGCGCCAGGACCTATGAG | CCCCTGGCTTTCGATTGGTT |
| Hoxa9 | GTCCCACGCTTGACACTCAC | CGGGTTATTGGGATCGATGGG |
| Hoxa10 | CCACCACCCACTCTGGTTTG | CCTTTGGAACTGCCCAGGGA |
| Hoxa11 | CGGTGGCTCCGGTGG | CTTGACGGTCGGTGAGGTTG |
